# *Crataegus monogyna* fruit aqueous extract as a protective agent against doxorubicin-induced reproductive toxicity in male rats

**Published:** 2013

**Authors:** Ali Shalizar Jalali, Shapour Hasanzadeh

**Affiliations:** 1 *Department of Basic Sciences, Faculty of Veterinary Medicine, Urmia University, Urmia, I. R. Iran*

**Keywords:** *Crataegus monogyna*, Doxorubicin, Reproductive Toxicity

## Abstract

**Objective: **Doxorubicin (DOX) is a broad spectrum chemotherapeutic agent used in the treatment of several malignancies. The use of DOX in clinical chemotherapy has been restricted due to its diverse toxicities, including reproductive toxicity.* Crataegus monogyna (C. monogyna) *is one of the oldest medicinal plants that have been shown to be cytoprotective because of scavenging free radicals. The present study was undertaken to determine whether *C. monogyna *fruits aqueous extract could serve as a protective agent against reproductive toxicity during DOX treatment in a rat model through antioxidant-mediated mechanisms.

**Materials and Methods: **Male Wistar rats were allocated to four groups. Two groups of rats were treated with DOX at a dose of 4 mg/kg intraperitoneally on days 1, 7, 14, 21, and 28 (accumulated dose of 20 mg/kg). One of the groups received *C. monogyna* fruits aqueous extract at a dose of 20 mg/kg per day orally for 28 days along with DOX. A vehicle-treated control group and a *C. monogyna* control group were also included.

**Results: **The DOX-treated group showed significant decreases in the body and organ weights and spermatogenic activities as well as many histological alterations. DOX treatment also caused a significant decrease in sperm count and motility with an increase in dead and abnormal sperms. Moreover, significant decrease in serum levels of testosterone and increased serum concentrations of FSH, LH, LDH, CPK, and SGOT were observed in DOX-treated rats. Notably, *Crataegus *co-administration caused a partial recovery in above-mentioned parameters.

**Conclusion: **These findings indicated that doxorubicin can adversely damage the testicular tissue, while *Crataegus *co-administration could effectively prevent these adverse effects by effective inhibiting oxidative processes and restoration of antioxidant defense system.

## Introduction

Doxorubicin (DOX), an anthracycline antibiotic isolated from the soil fungus *Streptomyces peucetius caesius*, is one of the most used anticancer drugs (Yeh et al., 2007a). It is recognized to be effective against many chemo-responsive tumors such as ovarian cancers, breast cancers, and lymphomas (Atessahin et al., 2006a ▶). Nonetheless, DOX is responsible for a wide range of adverse effects including reproductive toxicity in humans and experimental animals (Bechter et al., 1987 ▶; Damani et al., 2002 ▶). It has been shown that even a low dose of DOX (1 mg/kg b.w.) given to adult mice is able to target testicular germ cells, mainly A1-A4 spermatogonia, leading to seminiferous epithelium depletion (Lu and Meistrich, 1979 ▶). 

Moreover, it can also harm type B spermatogonia (Jahnukainen et al., 2000 ▶) and primary spermatocytes (Lu and Meistrich, 1979 ▶), induce germ cell apoptosis in testis (Hou et al., 2005 ▶), affect testicular lipids (Zanetti et al., 2007 ▶), and result in testicular failure eventually (Hacker-Klom et al., 1986 ▶). Furthermore, it has been reported that DOX causes decrease in weight of reproductive organs (Kang et al., 2002) and reduction of sperms concentration (Kato et al., 2001 ▶) and motility (Prahalathan et al., 2005a ▶). Although, the precise biochemical mechanism by which DOX causes testicular toxicity is still unclear, recent studies have suggested that DOX-induced organopathy involves the generation of reactive oxygen species (ROS), including O_2_^−•^, ^•^OH, and H_2_O_2_, which result in membrane and macromolecule damage by lipid peroxidation, DNA fragmentation, and protein oxidation (Xu et al., 2001 ▶; Quiles et al., 2002; Prahalathan et al., 2005b ▶). Therefore, DNA of rapidly dividing cells such as the testicular germ cells can be the preferential target of DOX. The drug intercalates within DNA strands causing cell cycle blockage in the G_2_ phase, single-strand breaks, and inhibition of the activity of some nuclear proteins, such as DNA and RNA-polimerase and DNA-topoisomerase II (Konopa, 1988 ▶; Speth et al., 1988 ▶). Furthermore, mammalian spermatozoa are particularly vulnerable to oxidative damage because of high concentration of polyunsaturated fatty acids and low antioxidant capacity (Vernet et al., 2004 ▶). Based on this concept, combination of the drug delivery together with potent and safe antioxidant may be the appropriate approach to ameliorate DOX-induced reproductive toxicity. 

Hawthorn (*Crataegus*), found in northern temperate regions such as East Asia, Europe, and Eastern North America, is a genus of the Rosaceae family. The two most commonly used species are *Crataegus laevigata *(syn *Crataegus oxyacantha*) and *Crataegus monogyna*. Hawthorn was first mentioned as a drug in the Tang-Ben-Cao (659 A.D.), which is the world’s earliest officially published pharmacopoeia (Yao et al., 2008 ▶). Independent studies have shown that extracts of *Crataegus *(from several parts of the plant including fruits) are rich in proanthocyanidins and flavonoids (Bahorun et al., 1996 ▶; Ljubuncic et al., 2005 ▶) and many of these phenolic compounds have been shown to be cytoprotective by scavenging superoxide anion, hydroxyl radical, hydrogen peroxides, and reducing lipid peroxidation (Bahorun et al., 1994 ▶; Zhang et al., 2001 ▶; Rice-Evans, 2004 ▶). In view of this, since the DOX-induced reproductive toxicity is linked to oxidative stress, the present study was undertaken to assess the possible protective effect of *C. monogyna *fruits aqueous extract with antioxidant properties against reproductive toxicity during DOX treatment in a rat model. 

## Materials and Methods

**Plant material**

The ripe fruits of *C. monogyna *were collected from its natural habitat around the city of Urmia in West Azerbaijan province, northwestern Iran. A dried voucher specimen was deposited at the Herbarium of the Botany Department, Faculty of Science, Urmia University under number 7031.

**Preparation of the aqueous extract**

After collection, the fruits were dried for 7–10 days in the shade at room temperature. Dried fruits were then ground and the powder was stored in cloth bags at 5 °C until transfer to the laboratory for extraction. The method for preparing dry water-soluble plant powders has been previously described (Ljubuncic et al., 2005 ▶). Briefly, dried plant material (25 g) was stirred in 250 mL of distilled water for 15 minutes at 100 °C, followed by rapid filtration through a crude cellulose filter and then Whatman #1 filter paper. The resulting filtrate was freeze-dried and the powder was stored at −18 °C in a desiccant until required. The average (w/w) yield was 12.4 %. 

**Animal model**

Adult sexually matured male (4 months of age weighing 176.70±6.23 g) albino rats of Wistar strain were obtained from Animal Resources Center of Veterinary Faculty of Urmia University. They were housed in a specific pathogen-free environment under standard conditions of temperature (25±2 °C), relative humidity (50±10%) and light (12 h light/12 h dark). They were fed with a standard pellet diet and had free access to water. Animals were checked daily for occurrence of any toxic signs. All ethical themes of the studies on animals were considered carefully and the experimental protocol was approved by the institute Review Board. 

**Experimental protocol**

After 7 days of acclimation to the environment, the rats were randomly divided into four groups of six animals each (*n*=6): control group (Control), *Crataegus* group (*Crataegus*), doxorubicin group (DOX), and doxorubicin-*Crataegus *group (DOX+*Crataegus*). The two experimental groups (DOX and DOX+*Crataegus*) were treated with DOX (Ebedoxo®, EBEWE Pharma Ges.m.b.H. Nfg. KG, Austria) at a dose of 4 mg/kg BW intraperitoneally on days 1, 7, 14, 21, and 28 (accumulated dose of 20 mg/kg). The group (*Crataegus*) was gavaged *C. monogyna *aqueous extract at a dose of 20 mg/kg b.w. per day for 28 days. The (DOX+*Crataegus*) group also received the same dose of the extract along with DOX administration. The controls were treated with distilled water (5 ml/kg b.w., p.o.) for 28 days and injected with normal saline (2 ml/kg, i.p.) on days 1, 7, 14, 21, and 28. The treatment period was 28 days. The protocol for this study, including doses and duration of treatment for DOX and *Crataegus*, was designed according to previous studies (Khalil et al., 2008 ▶; Patil and Balaraman, 2009 ▶; Saalu et al., 2010a ▶).

**Sampling**

Animals were euthanized by CO_2_ exposure in a special device following anesthesia with ketamine (75 mg/kg, i.p.) 24 hours after the last treatment. Blood was collected without anticoagulant for serological analyses. Testes, epididymides, and accessory sex glands were quickly dissected out, cleared of adhering connective tissue, and were weighed on a Mattler Basbal scale (Delta Range, Tokyo). Testes were freshly cut with frozen section and periodic acid shiff (PAS) special staining technique was conducted for histological evaluation. 

**Sperm characteristics**

In order to assess the sperm motility, one caudal epididymis was placed in 1 mL of Ham’s F10 medium. Cauda was cut into 2–3 pieces and incubated at 37 °C for 10 minutes in CO_2_ incubator to allow sperm to swim out of the epididymal tubules. One drop of sperm suspension was placed on a microscope slide, and a cover slip was placed over the droplet. At least 10 microscopic fields were observed at 400× magnification using a phase contrast microscope and the percentage of motile sperm was evaluated microscopically within 2–4 minutes of their isolation from the epididymides and was expressed as a percentage of motile sperm of the total sperm counted (Selvakumar et al., 2006 ▶).

The epididymal sperm count was determined by hemocytometer. After dilution of epididymal sperm to 1:20 in Ham’s medium, approximately 10 μL of this diluted specimen was transferred to each of the counting chambers of the hemocytometer, which was allowed to stand for 5 minutes in a humid chamber to prevent drying. The cells sedimented during this time and were counted with a light microscope at 400×. The sperm count was expressed as number of sperm per milliliter (Zambrano et al., 2005 ▶). 

A 20 μL of sperm suspension was mixed with an equal volume of 0.05% eosin-Y. After 2 minutes incubation at room temperature, slides were viewed by bright-field microscope with 400× magnification. Dead sperms appeared pink and live sperms were not stained. Two hundred sperms were counted in each sample and viability percentages were calculated. For the analysis of morphological abnormalities, sperm smears were drawn on clean and grease-free slides, and allowed to dry in air overnight. The slides were stained with 1% eosin-Y/5 % nigrosin and examined at 400× for the presence of morphological abnormalities such as amorphous, hook less, bicephalic, coiled, or abnormal tails (Wyrobek et al., 1983 ▶).

**Biochemical parameters**

Serum concentrations of follicle-stimulating hormone (FSH) and luteinizing hormone (LH) were measured by enzyme-linked immunosorbent assay (ELISA) as described in the instructions provided by manufacturer’s kits (Monobind Inc., USA) as well as testosterone (Demeditec Diagnostics GmbH, Germany). The activities of serum lactate dehydrogenase (LDH), creatine phosphokinase (CPK), and glutamic oxaloacetate transaminase (SGOT) were measured using an automatic blood chemistry analyzer (BT3000 Plus, Biotecnica Instruments, Italy). 

**Histological parameters**

For each testis, five vertical sections from the polar and the equatorial regions were sampled (Qin and Lung, 2002 ▶) and an unbiased numerical estimation of the following histological parameters was determined using a systematic random scheme. 

**Tubule differentiation index (TDI) and spermiation index (SPI)**

Two hundred cross-sections of seminiferous tubules were randomly analyzed in each rat (one hundred per testis) for the calculation of tubule differentiation index (TDI) and spermiation index (SPI). TDI is the percentage of seminiferous tubules containing at least three differentiated germ cells (Porter et al., 2006 ▶). SPI is the percentage of seminiferous tubules with normal spermiation (Rezvanfar et al., 2008 ▶).

**Statistical analysis**

Results are expressed as mean±SD. Differences between groups were assessed by the analysis of variance (ANOVA) using the SPSS software package for Windows. Statistical significance between groups was determined by Tukey’s multiple comparison post-hoc test and the p-values less than 0.05 were considered to be statistically significant. 

## Results

**Clinical signs and body and organ weight changes**

All animals survived the experimental period. DOX-treated animals showed general signs of deterioration such as piloerection, alopecia, lethargy, hunched posture, shivers, sedation, and low activity. The final body weight, absolute weight of testes and epididymides, and relative weight of testes as well as seminal vesicles and ventral prostate weights were significantly lower than those of the controls after DOX treatment, whereas daily administration of *Crataegus *caused significant increase in the final body weight, absolute and relative weights of testes, absolute weight of epididymides and seminal vesicles and ventral prostate weights of doxorubicin-*crataegus* group in comparison with DOX group. Absolute and relative weights of testes and absolute weight of epididymides as well as seminal vesicles weight increased significantly in *Crataegus *group compared with the control (Table 1). 

**Sperm characteristics**

Treatment of male rats with DOX caused a significant decrease in the sperm concentration and motility, while dead and abnormal sperms increased compared with those of the control (Table 2). Co-administration of *C. monogyna *fruits aqueous extract caused a significant increase in semen quality and minimized toxic effects of DOX. 

**Biochemical findings**

Administration of DOX alone significantly increased serum level of CPK, LDH, and SGOT compared with the control rats (Table 3). Moreover, serum concentrations of FSH and LH were significantly elevated, while serum level of testosterone decreased by DOX treatment (Table 4). The administration of *C. monogyna *fruits aqueous extract along with DOX significantly restored serum marker levels towards the control value. 

**Table 1:** Effect of doxorubicin and *Crataegus monogyna *fruits aqueous extract on body weight and weights of testis, epididymis, seminal vesicles, and ventral prostate

	Control	DOX	*Crataegus*	DOX + *Crataegus*
Final Body Weight (BW, g)	225.66±3.20	160.83±5.63^a^	228.50±3.72^b^	189.16±4.87^a^,^b^
Absolute weight (g)				
Testes	2.00±0.062	1.31±0.046^a^	2.13±0.012 a,b	1.60±0.058 a,b
Epididymides	1.14±0.035	0.83±0.022^a^	1.21±0.012 a,b	1.00±0.028 a,b
Relative weight (per BW, %)				
Testes	0.88±0.017	0.81±0.012^a^	0.93±0.010 a,b	0.84±0.017 a,b
Epididymides	0.50±0.018	0.51±0.024	0.52±0.005	0.53±0.017
Seminal vesicles (mg)	644.50±36.16	436.50±21.74^a^	737.33±32.37 a,b	526.33±44.49 a,b
Ventral Prostate (mg)	191.66±6.94	163.66±8.45^a^	191.50±12.81^b^	180.33±10.76^b^

The values are expressed as mean±SD (n = 6).

(a) Significant differences as compared with the control group at p*<*0*.*05,

(b) Significant differences as compared with the doxorubicin group at p*<*0*.*05.

**Table 2:** Effect of doxorubicin and *Crataegus monogyna *fruits aqueous extract on epididymal sperm characteristics

	Control	DOX	*Crataegus*	DOX+*Crataegus*
**Sperm count (10** ^6^ **/ml)**	77.66±11.94	14.50±4.46^a^	72.16±5.45^b^	51.00±5.65^a^,^b^
**Motility (%)**	82.35±1.64	42.32±1.14^a^	82.10±1.18^b^	52.92±1.81 a,b
**Dead sperms (%)**	8.95±0.62	45.83±1.71^a^	8.62±0.41^b^	28.04±1.27 a,b
**Abnormal sperms (%)**	7.70±0.78	31.33±2.47^a^	7.41±0.86^b^	20.70±1.16 a,b

The values are expressed as mean±SD (n = 6).

(a) Significant differences as compared with the control group at p*<*0*.*05,

(b) Significant differences as compared with the doxorubicin group at p*<*0*.*05.

**Table 3:** Effect of doxorubicin and *Crataegus monogyna *fruits aqueous extract on serum lactate dehydrogenase (LDH), creatine phosphokinase (CPK), and glutamic oxaloacetate transaminase (SGOT) activities

	Control	DOX	*Crataegus*	DOX+*Crataegus*
**LDH (IU/l)**	281.66±15.98	631.16±54.71^a^	256.16±52.25^b^	484.83±38.66^a^,^b^
**CPK (IU/l)**	245.50±11.70	607.83±14.53^a^	251.66±8.89^b^	520.50±12.84 a,b
**SGOT (IU/l)**	94.50±10.25	203.16±12.60^a^	97.16±13.90^b^	182.00±9.31 a,b

The values are expressed as mean±SD (n = 6).

(a) Significant differences as compared with the control group at p*<*0*.*05,

(b) Significant differences as compared with the doxorubicin group at p*<*0*.*05.

**Table 4:** Effect of doxorubicin and *Crataegus monogyna *fruits aqueous extract on serum concentrations of sex hormones.

	Control	DOX	*Crataegus*	DOX+*Crataegus*
**FSH (mIU/ml)**	0.24±0.03	0.52±0.05^a^	0.28±0.03^b^	0.39±0.05^a^,^b^
**LH (mIU/ml)**	0.29±0.02	0.63±0.05^a^	0.31±0.03^b^	0.52±0.07 a,b
**Testosterone (ng/ml)**	6.85±0.39	3.16±0.22^a^	6.63±0.48^b^	4.82±0.30 a,b

The values are expressed as mean±SD (n = 6).

(a) Significant differences as compared with the control group at p*<*0*.*05,

(b) Significant differences as compared with the doxorubicin group at p*<*0*.*05.

**Table 5:** Effect of doxorubicin and *Crataegus monogyna *fruits aqueous extract on tubule differentiation index (TDI) and spermiation index (SPI).

	Control	DOX	*Crataegus*	DOX+*Crataegus*
**TDI (%)**	92.33±2.46	16.83±2.97^a^	92.75±3.31^b^	63.58±4.87^a^,^b^
**SPI (%)**	91.25±1.44	14.16±2.13^a^	91.58±3.26^b^	59.50±4.43^a^,^b^

The values are expressed as mean±S.D. (n = 6)

(a) Significant differences as compared with the control group at p*<*0*.*05,

(b) Significant differences as compared with the doxorubicin group at p*<*0*.*05.

**Histopathologic findings**

**Figure 1:**
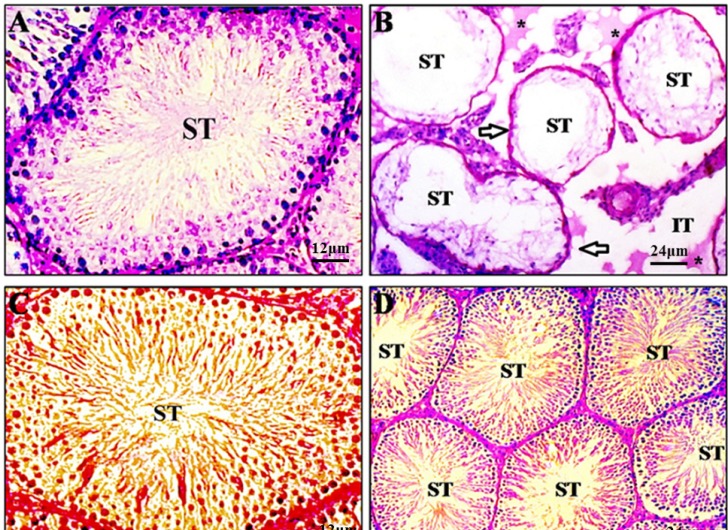
Photomicrographs of testicular sections of control (A), doxorubicin (B), *Crataegus* (C), and doxorubicin+*Crataegus *(D) treated rats. Testes from control group exhibit a normal feature of seminiferous epithelium (ST) and interstitial tissue (IT) with active spermatogenesis (A) as well as *Crataegus*-treated rats (C). However, a testis from a doxorubicin treated rat reveals markedly shrunken seminiferous tubules with severe germ cell aplasia and basement membrane thickening (arrows). Note Rupture, vacuolization, oedematous fluid accumulation (asterisks), and interstitial space widening in intertubular connective tissue (B). *Crataegus* co-treated animals display nearly normal histoarchitecture (D). Periodic acid shiff staining method.

DOX induced drastic morphologic changes in the testis (Figure 1B). Shrunken seminiferous tubules showed severe germ cell aplasia and basement membrane thickening as well as rupture, vacuolization, edematous fluid accumulation, and fibrosis in interstitial and peritubular tissue. In these specimens, Leydig cells were degenerated and appeared with pyknotic nuclei. Moreover, Sertoli cells lost their junction with germ cells and looked amorphous with irregular and smaller nuclei. Administration of *Crataegus *along with DOX restored these changes towards normality (Figure 1D). 

**Histological parameters**

As seen in Table 5, DOX induced deletion of germ cells during spermatogenesis, which resulted in a dramatic decrease in TDI. Due to the germ cells deletion, the SPI was greatly decreased in the DOX-treated animals. *Crataegus *co-administration significantly attenuated the DOX–induced germ cell loss from seminiferous tubules.

## Discussion

Testicular dysfunction is amongst the most common long-term side effects of cytotoxic chemotherapy used in the treatment of many malignancies. In a clinical context, testicular germinal epithelium damage in patients exposed to chemotherapeutic agents could result in long-term male infertility or genetic alterations (Howell, and Shalet, 2001). A strategy to decrease the incidence of serious side-effects of anticancer drugs with preservation of their chemotherapeutic efficacy is necessary. The clinical application of DOX has been largely complicated by its potential toxicity to the various organs including testis (Imahie et al., 1995 ▶; Kato et al., 2001 ▶). The DOX-induced testicular cytotoxicity appears to be mainly due to overload of oxidative stress, breakage of DNA continuity, and induction of cell apoptosis (Atessahin et al., 2006b ▶).

 In the present study, reduction in body weight, weight of the testis, epididymis, and accessory sex glands, and histological changes in testis were indicative of drug toxicity. Since the weight of the testes largely depends on the mass of the differentiated spermatogenic cells (Katoh et al., 2002 ▶), the marked reduction in organ weight by DOX can be explained by diminished number of germ cells, atrophy of Leydig cells, and a significant lower rate of spermatogenesis as confirmed by our findings. Reduction in the weight of testes and accessory reproductive organs in DOX-treated animals reflect the reduced availability of androgens (Patil et al., 1998 ▶). Increased generation of free radicals is one of the possible mechanisms involved in chemotherapeutic agents-induced Leydig cell degeneration which result in marked reduction of serum testosterone (Debnath and Mandal, 2000 ▶). Moreover, significant increase in serum LH levels certainly indicates disturbance in Leydig cell function (Jequier, 2000 ▶). Chemotherapy can result in long-term or permanent azoospermia, the mechanism of which is most likely the death of germ cells (Meistrich, 1986 ▶). Histological parameters such as tubule differentiation index and spermiation index can also give information about the testicular damage degree as a consequence of germ cell death. In general, massive germ cell loss caused by anticancer drugs is followed by a sharp decline in testicular histological parameters (França and Russell, 1998 ▶). As shown in the present study, depletion of seminiferous epithelium and the consequent decrease of histological measurements caused by cytotoxic agents were confirmed in our report.

Structural development and maturation of germ cells and spermiation are important functions of Sertoli cells (Mruk and Cheng, 2004 ▶). Therefore, a potential explanation for the failure of spermatogenesis in the DOX-treated males is disruption of testosterone-dependent junction of Sertoli cerlls with germ cells leading to their disorganization and separation. Additionally, FSH elevation can be an indication of spermatogenesis failure related to various causes including: testicular failure, genetic abnormalities, and toxic exposure such as radiation, chemotherapy, and heat (Lewis, 2007 ▶). Moreover, it indicates the abnormal Sertoli cell function resulting in reduced inhibin secretion (Bergmann et al., 1994).

 In the present study, epididymal sperm count and motility decreased by DOX treatment while the number of dead and abnormal sperms increased, confirming a previous report that DOX provokes disruption of spermatogenic cells maturation, epididymis sperm concentration reduction, and alteration of sperm morphology (Meistrich et al., 1990 ▶). The decreased sperm count clearly shows the elimination of sperm cells at different stages of development and points to free radical attack through DOX metabolism. In fact, oxidative damage to polyunsaturated fatty acids of cell membranes has long been considered to result in the impairment of membrane fluidity and permeability. This results in the damage of germ cells, spermatozoa, and mature sperm (Sikka, 2004 ▶). It has also been reported that DOX causes an increase in apoptosis of meiotically dividing spermatocytes and type A and intermediate spermatogonia (Shinoda et al., 1999 ▶) by intercalating into DNA to produce strand breaks and by inhibiting topoisomerase II activity (Myers and Chabner, 1990 ▶). Hence, the decrease in epididymal sperm count observed in DOX-treated rats might reflect the enhanced destruction and/or reduced production of spermatogenic cells. The significant reduction in sperm motility may be due to the toxic effect of cytotoxic drugs on the sperm flagellum through rapid loss of intracellular ATP (Rezvanfar et al., 2008 ▶). 

It has been shown that chemotherapy can result in the decrease of testicular tricarboxylic acid cycle enzyme activities and thus energy metabolism impairment (Selvakumar et al., 2005 ▶). Since ATP may serve as an energy source for sperm motility, the decrease in energy metabolism may play a crucial role in the loss of sperm motility in DOX-administered rats. Mammalian spermatozoa are exceptionally susceptible to damage from reactive oxygen species because of their fragile characteristics in response to oxidative stress (Rao et al., 1989 ▶). Therefore, oxidative stress could result in sperms viability and integrity disturbance through lipid peroxidation, DNA fragmentation, and protein oxidation (Sikka, 1996 ▶; Agarwal and Saleh, 2002 ▶). 

In our study, LDH, CPK, and SGOT activities in serum were significantly elevated. These findings suggest that DOX may induce generalized toxicity in rats. To date, a number of studies have shown benefits of antioxidants in protecting male reproductive system from deleterious effects of reactive oxygen species and other free radicals generated during DOX exposure. It was found that ginseng intestinal metabolite-I (GIM-I), the final intestinal bacterial metabolite of ginseng in humans with antioxidant effects, ameliorates DOX-induced reproductive toxicity (Kang et al., 2002*)* as well as doxycycline (Yeh et al., 2007b). There is also evidence that green tea extracts can attenuate DOX-induced spermatogenic disorders in conjunction with higher telomerase activity levels (Sato et al., 2010). Recently, two studies also have indicated that grapefruit seed extract, an antioxidant-rich compound, reduces DOX-induced reproductive toxicity through reduction of oxidative stress (Saalu et al., 2010a ▶; Saalu et al., 2010b ▶).

 In the present study, it has been shown that *Crataegus monogyna* fruits aqueous extract co-administration was effective in protection or attenuation of testicular damage following DOX exposure. Increasing evidences support the fact that* Crataegus* is beneficial where free radicals are known to play a predominant role in toxicity. Previous studies have shown that hawthorn extract can effectively lessen the extent of oxidative stress induced by cyclophosphamide in mouse bone marrow cells due to its strong antioxidant activity (Hosseinimehr et al., 2008 ▶). Furthermore, it has been revealed that hawthorn extract reduces infarct volume and improves neurological score by reducing oxidative stress in rat brain (Elango et al., 2009 ▶). In conclusion, the findings of our study indicate that DOX can adversely damage the testicular tissue by imposing oxidative stress, while *Crataegus monogyna* fruits aqueous extract co-administration could effectively counteract DOX-related oxidative injury to testicular tissue through restoration of antioxidant defense system.
